# Whole Genome Sequencing and Morphological Trait-Based Evaluation of UPOV Option 2 for DUS Testing in Rice

**DOI:** 10.3389/fgene.2022.945015

**Published:** 2022-08-26

**Authors:** Hong Liu, Dehua Rao, Tao Guo, Sunil S. Gangurde, Yanbin Hong, Mengqiang Chen, Zhanquan Huang, Yuan Jiang, Zhenjiang Xu, Zhiqiang Chen

**Affiliations:** ^1^ National Engineering Research Center of Plant Space Breeding, South China Agricultural University, Guangzhou, Guangdong, China; ^2^ College of Agriculture, South China Agricultural University, Guangzhou, Guangdong, China; ^3^ International Crops Research Institute for the Semi-Arid Tropics (ICRISAT), Hyderabad, India; ^4^ Crop Protection and Management Research Unit, USDA-ARS, Tifton, GA, United States; ^5^ Department of Plant Pathology, University of Georgia, Tifton, GA, United States; ^6^ Guangdong Provincial Key Laboratory for Crops Genetic Improvement, Crops Research Institute, Guangdong Academy of Agricultural Sciences, Guangzhou, China

**Keywords:** rice, genotype, phenotype, SNP, correlation analysis, DUS, distinctness, genomic prediction

## Abstract

To evaluate the application potential of high-density SNPs in rice distinctness, uniformity, and stability (DUS) testing, we screened 37,929 SNP loci distributed on 12 rice chromosomes based on whole-genome resequencing of 122 rice accessions. These SNP loci were used to analyze the DUS testing of rice varieties based on the correlation between the molecular and phenotypic distances of varieties according to UPOV option 2. The results showed that statistical algorithms and the number of phenotypic traits and SNP loci all affected the correlation between the molecular and phenotypic distances of rice varieties. Relative to the other nine algorithms, the Jaccard similarity algorithm had the highest correlation of 0.6587. Both the number of SNPs and the number of phenotypes had a ceiling effect on the correlation between the molecular and phenotypic distances of varieties, and the ceiling effect of the number of SNP loci was more obvious. To overcome the correlation bottleneck, we used the genome-wide prediction method to predict 30 phenotypic traits and found that the prediction accuracy of some traits, such as the basal sheath anthocyanin color, glume length, and intensity of the green color of the leaf blade, was very low. In combination with group comparison analysis, we found that the key to overcoming the ceiling effect of correlation was to improve the resolution of traits with low predictive values. In addition, we also performed distinctness testing on rice varieties by using the molecular distance and phenotypic distance, and we found that there were large differences between the two methods, indicating that UPOV option 2 alone cannot replace the traditional phenotypic DUS testing. However, genotype and phenotype analysis together can increase the efficiency of DUS testing.

## Introduction

Rice (*Oryza sativa* L.) is one of the most important staple food crops for half of the population across the world (Hu et al., 2002). Rice production in China accounts for about 28.22% of the world’s total production (Food and Agriculture Organization, 2020). China is not only the largest rice producer and consumer, but it also has advanced rice breeding techniques and plenty of rice varieties. As of 1 April 2021, China had a total of 10,702 certified rice varieties, of which 3,243 varieties were under the protection of plant variety rights (Ministry of Agriculture and Rural Affairs of People’s Republic of China, 2021).

Distinctness, uniformity, and stability (DUS) are the basic requirements for the certification of a plant variety. In the DUS testing process, uniformity testing and stability testing are the foundation and distinctness testing is the core. Currently, rice DUS testing requires at least two independent growth cycles as per the standard DUS testing protocols. The current DUS testing is only based on phenotypic trait analysis. Although morphological analysis is very direct, it is easily affected by environmental conditions. The phenotypes of the same variety may vary significantly depending on time and location; in addition, morphological analysis is time consuming, laborious, and inefficient. Moreover, as a result of the consistent breeding goals, breeders often use backbone parents with the similar genetic relationships for cross-breeding, which results in low genetic diversity of the bred varieties (Liu and Zhang, 2010) and brings challenges for phenotypic testing. However, in comparison with morphological characteristics, molecular markers can be used at various developmental stages and they are not affected by the environment. Moreover, molecular markers are abundant and genetically stable, and they are most widely used for genetic diversity analyses in almost all crops (Hayward et al., 2015; Hong et al*.,* 2021). Molecular markers have diverse applications in breeding programs, such as F1 confirmation, cultivar/hybrid purity testing, DNA finger printing (Gangurde et al., 2017), foreground and background selection (Shasidhar et al., 2020), marker-assisted selection (Cockram et al., 2012; Wagh et al., 2021), and genetic mapping (Dodia et al., 2019; Jadhav et al., 2021). Molecular markers are also widely used in the identification of rice varieties.

Pourabed et al. (2015) reported that SSR markers could assist in rice DUS testing. Zheng et al. (2022) reported that 40 SNP markers could be used to successfully discriminate between *indica* and *japonica* rice, with a correlation coefficient of 0.86 with Cheng’s index method. Steele et al. (2021) developed a set of KASP markers for rapid genotyping and identification of basmati rice varieties.

The International Union for the Protection of New Varieties of Plants (UPOV) also proposed three options to incorporate molecular marker technology into DUS testing (UPOV INF/18/1, 2011): prediction of phenotypic characteristics by using linked diagnostic markers (option1); calculation of molecular distance thresholds to reproduce phenotypic distinctness determination (option 2); and use of an unlimited number of molecular markers to reconstruct a new test system (option 3). In the case of option 1, because the current development of diagnostic markers for rice mainly focuses on important agronomic traits, such as yield, quality, and disease resistance, and there are few additional studies on other non-major agronomic traits, there are not enough diagnostic markers to evaluate and analyze this option. In the case of option 3, there is also much controversy because setting the threshold for determination of distinctness at 1 base pair difference could lead to impractical determination in uniformity and stability testing. Currently, research into rice DUS testing is mainly focused on option 2, which is based on a high correlation between the molecular and phenotypic distances of varieties. Previous studies have conducted in-depth research on option 2. By using 3,072 SNP markers for barley variety-distinct analysis, Jones et al. (2013) found that the correlation between the molecular and phenotypic distances of barley varieties was between 0.557 and 0.637. It was also believed that the correlation was affected by kinship and the number of molecular markers. Liu et al. (2019) used morphological traits and SSR markers to analyze the genetic diversity of peanut varieties and found that the correlation between them was 0.36. Guan et al. (2020) used 384 SNPs to perform maize DUS testing and found that the correlation was only 0.21. The results of previous studies showed that the correlations between phenotypic distances and molecular distances were generally not significantly high, which directly affected the application of UPOV option 2.

With the advances in sequencing technologies and the reduction of sequencing costs, SNP markers have become popular molecular markers in genome research. They have been widely used in genetic structure analysis (Ebana et al., 2010), genome-wide association analysis (Huang et al., 2012; Gangurde et al., 2020; Pujar et al., 2020; Wang et al., 2020), and genome-wide selection (Cui et al., 2020). Compared with SSR markers, SNPs have the advantages of genome-wide distribution and high density, and they are more suitable for efficient automated analysis. In the present study, on the basis of whole-genome sequencing of 122 rice accessions, we analyzed the correlation between the molecular and phenotypic distances of rice varieties by screening 37,929 SNP loci, and we also evaluated UPOV option 2 for the DUS testing of rice.

## Materials and Methods

### Experimental Materials

A total of 122 *japonica* rice varieties, most from China and Japan (Table 1), were used as the main experimental materials. These rice varieties were provided by the China National Rice Research Institute and the National Engineering Research Center of Plant Space Breeding of South China Agricultural University. The varieties selected for this study contained both elite lines and landraces, as well as breeding lines, some of which were sister lines. As all varieties were phenotypically distinct from each other, these varieties were suitable to evaluate UPOV option 2 for the DUS testing of rice.

**TABLE 1:** Rice accessions used in the present study.

No.	Cultivator	Country	No.	Cultivator	Country	No.	Cultivator	Country
A1	Tailangpingweiju	Thailand	A42	Nonglin-9	Japan	A83	Chaozhiguang	Japan
A2	Boyo	Indonesia	A43	Beinian	Japan	A84	Aizhi-78	Japan
A3	RT103	Congo	A44	Qingfeng	Japan	A85	Aizhi-80	Japan
A4	UDEK	Indonesia	A45	Libingnuo	Japan	A86	Guandong-11	Japan
A5	Xiaohongnuo	China	A46	Dongfengnian-17	Japan	A87	Nonglin-93	Japan
A6	Huangsinuo	China	A47	Fangzhu-5	Japan	A88	Fengguang	Japan
A7	Cungunuo	China	A48	Xinrong	Japan	A89	Yueguang	Japan
A8	Zaonuo	China	A49	Nonglin-268	Japan	A90	Yusuibo	Japan
A9	Gaogannuo	China	A50	Kujiuwang-3	Japan	A91	Xushi	Japan
A10	Baokanglengshuihong	China	A51	Yulong	Japan	A92	Misui	Japan
A11	Zaohongnuo	China	A52	Guijin	Japan	A93	Youjinjin	Japan
A12	Sanlicun	China	A53	Songmunuo	Japan	A94	Aizhi-53	Japan
A13	JR7729-2	Philippines	A54	Suwecn	Japan	A95	Nonglin-288	Japan
A14	JW60	India	A55	Jisa-1	Japan	A96	Nonglin-218	Japan
A15	Erbaixuan	China	A56	Tengban-4	Japan	A97	Bifeng	Japan
A16	Lengshuibai	China	A57	Aoyu-187	Japan	A98	Guobao-P2	Japan
A17	Lengshuinuo	China	A58	Yuxingnuo	Japan	A99	Rizhiguang	Japan
A18	Bingshuibai	China	A59	Xinfangjiu-4	Japan	A100	Yuanye-4	Japan
A19	Jiuyuehuang-1	China	A60	Weihuamin-2	Japan	A101	Nanjingnongda-W30	China
A20	Zhangdianzaonuogu	China	A61	Qingxinuo-107	Japan	A102	Nanjing-16	China
A21	Kawluyoeng	Thailand	A62	Chaofeng-1	Japan	A103	Milyang63	South Korea
A22	Dongnong-363	China	A63	Nonglin-276	Japan	A104	Omc-9	Vietnam
A23	Zixiangnuo	China	A64	Qiutianxiaodin	Japan	A105	Duzi-129	Soviet Union
A24	Yuanzizhandao	China	A65	Xinan-72	Japan	A106	Chendao	China
A25	Yuli	Japan	A66	Feiqinian	Japan	A107	Bodao-1	China
A26	Hejiang-18	China	A67	Sanliyannuo	Japan	A108	Bodao-2	China
A27	Hanlundao	China	A68	Nonglin-277	Japan	A109	Aenmetan-2	Indonesia
A28	Guihuahuang	China	A69	Aoyu-324	Japan	A110	Salazana-3	Madagascar
A29	Tsukushiakamochi	Japan	A70	Luyu-42	Japan	A111	Xuelihong	China
A30	Koshihikari	Japan	A71	Aoyu-191	Japan	A112	Heimi-2	China
A31	Heuknambyeo	South Korea	A72	Nonglin-289	Japan	A113	Xinyidanuo	China
A32	Beniroman	Japan	A73	Xiaobei	Japan	A114	Hainanhong	China
A33	Asamurasaki	Japan	A74	Xiannan-1	Japan	A115	Hainannuo	China
A34	Lemont	United States	A75	Aoyu-334	Japan	A116	Hainanhei	China
A35	Aizhixiang	Japan	A76	Nonglin-285	Japan	A117	HN-27	China
A36	Ludaononglin-2	Japan	A77	Dadao	Japan	A118	HN-10	China
A37	Chunnuo	Japan	A78	Fengxu	Japan	A119	HN-54	China
A38	Youliujiannuo	Japan	A79	Luorongdao	Japan	A120	HN-107	China
A39	Fenghei	Japan	A80	Changqi	Japan	A121	HN-31	China
A40	Ludaononglinnuo-1	Japan	A81	Aoyu-2	Japan	A122	HN-61	China
A41	Ludaononglinnuo-21	Japan	A82	Xingnian	Japan			

### Extraction of Genomic DNA and SNP Calling

In this study, 30 plump seeds per accession were selected, sterilized with 1% sodium hypochlorite for 10 min, and then reconstituted three times with distilled water. The sterilized seeds were placed in germination bottles, an appropriate amount of distilled water was added, and then the bottles were placed in a germination box at 28°C for 14 days. High-quality genomic DNA was then extracted from 25 seedlings of each line by using a plant genomic DNA extraction kit (TIANGEN, China), and the quality was checked on a Nano-drop spectrophotometer. A Covaris sonicator was used to break the qualified DNA samples into approximately 350-bp fragments. An NEB Next® Ultra DNA Library Prep Kit (NEB, United States) was then used to prepare a DNA library, which included the processes of end repair, polyA tail addition, and ligation of adapter. Finally, the constructed library was sequenced with an Illumina NovaSeq PE150 sequencer at a sequencing depth of 10×. According to the alignment results of sequencing data on the rice reference genome (MSU-RGAP 7.0), SNPs were called by using the GATK software toolkit (McKenna et al., 2010). Furthermore, VCFtools software (Danecek et al., 2011) was used to filter 738,341 SNPs with a minimum allele frequency (MAF) greater than 0.05 and missing rate less than 0.2. Finally, after comparing these SNPs with the 3K rice core SNPs (The 3K RGP, 2014), we selected a total of 37,929 SNPs (Supplementary Table S1) to evaluate UPOV option 2 in rice.

### Morphological Survey

The experimental rice varieties were planted during September–December 2021 at the Wushan experimental base of South China Agricultural University, according to the requirements of the UPOV test guide for rice (UPOV TG/16/8, 2004; UPOV TG/16/9, 2020). Each plot was 1.5 m long and 1 m wide, with a row spacing of 20 cm and a plant spacing of 10 cm. Phenotypic data were recorded for 30 morphological traits (Table 2; Supplementary Table S2). Among them, visual traits were investigated by inspection and recorded with grade codes 1–9, and quantitative traits were measured with scale tools and converted into grade codes 1–9 based on standard varieties (UPOV TG/16/8, 2004; UPOV TG/16/9, 2020).

**TABLE 2:** 30 Morphological traits used for the DUS testing of rice.

No.	Trait	No.	Trait
1	Basal leaf sheath: anthocyanin coloration	16	Panicle: exsertion
2	Plant: growth habit	17	Glume: length
3	Leaf blade: intensity of green color	18	Lemma: color
4	Leaf blade: anthocyanin coloration	19	Grain: ratio length/width
5	Leaf blade: pubescence	20	Grain: color
6	Time of panicle emergence	21	Grain: aroma
7	Awn: length	22	Plant: number of panicles
8	Lemma: color of tip	23	Stem: thickness
9	Stigma: color	24	Stem: length
10	Stem: anthocyanin coloration of nodes	25	Flag leaf: length of blade
11	Lemma: pubescence	26	Flag leaf: width of blade
12	Flag leaf: attitude of blade	27	Panicle: length
13	Panicle: attitude	28	1000 seed weight
14	Panicle: number of secondary branches	29	Grain: length
15	Panicle: attitude of branches	30	Grain: width

### Statistical Analysis

In this study, Admixture software (Alexander et al., 2009) was used to analyze the population structure of accessions based on 37,929 SNPs. First, the number of clusters K of the tested materials was set to be 1–10, and then the cross-validation error (CVE) rate for each number of clusters was calculated. Finally, the K value corresponding to the minimum cross-validation error rate was determined as the optimal number of clusters. Principal component analysis (PCA) was performed by using the GCTA software (Yang et al., 2011), first by using the parameter “--make-grm” to obtain a genetic relationship matrix (GRM) and then by performing a plot analysis based on the first two principal components.

A phenotype 0–1 matrix was constructed based on investigation data of morphological traits. The variety that occurred on the level i of a trait was recorded as 1; otherwise, it was recorded as 0. Similarly, the SNP 0–1 matrix of rice varieties was constructed based on the SNP loci information, and the missing loci were filled with mode. Loci with the same information as the reference genome were marked as 0; otherwise, they were marked as 1, and heterozygous loci were marked as 0.5. R software (R Core Team, 2012) was used for statistical analysis. Initially, the Euclidean, Manhattan, Gower, Canberra, Harmonic_mean, Jaccard, Squared_euclidean, Person, Cosine, and Dice distances of morphological traits and SNP loci were calculated with the R package “philentropy” (Drost, 2018). Furthermore, the correlation between molecular and phenotypic distances of rice varieties was calculated, and the optimal similarity algorithm was screened. On the basis of the above analysis, the effect of trait number and SNP loci number on the correlation was analyzed. Then, 10%, 20%, 40%, 60%, 80%, and 100% of phenotypic distances were set as the thresholds to compare with the corresponding molecular distances (Jones et al., 2013), and UPOV option 2 was evaluated according to the efficiency of reproducibility. In this study, the R package “dendextend” (Galili, 2015) was used to analyze the phenotypic and molecular clustering results.

The rrBLUP (ridge regression best linear unbiased prediction) data package (Endelman, 2011) was used to perform genome-wide prediction analysis on 30 DUS traits based on 37,929 SNP loci. The formula is y=μ+Xα+e , where y is the best linear unbiased predictor vector for the trait of the tested variety, *µ* is the population mean, *α* is the additive effect of the markers, X is the genotype matrix, and *e* is the residual term. The training group comprised 90 randomly selected varieties, and the remaining 32 varieties were used as the testing group. The analysis was performed 100 times to calculate the prediction value of each trait. The correlation coefficient between the predicted value and the actual observed value for the trait in the testing group was used as the prediction accuracy.

## Results

### Analysis of Sequencing Results and Distribution of SNP Loci

**FIGURE 1:**
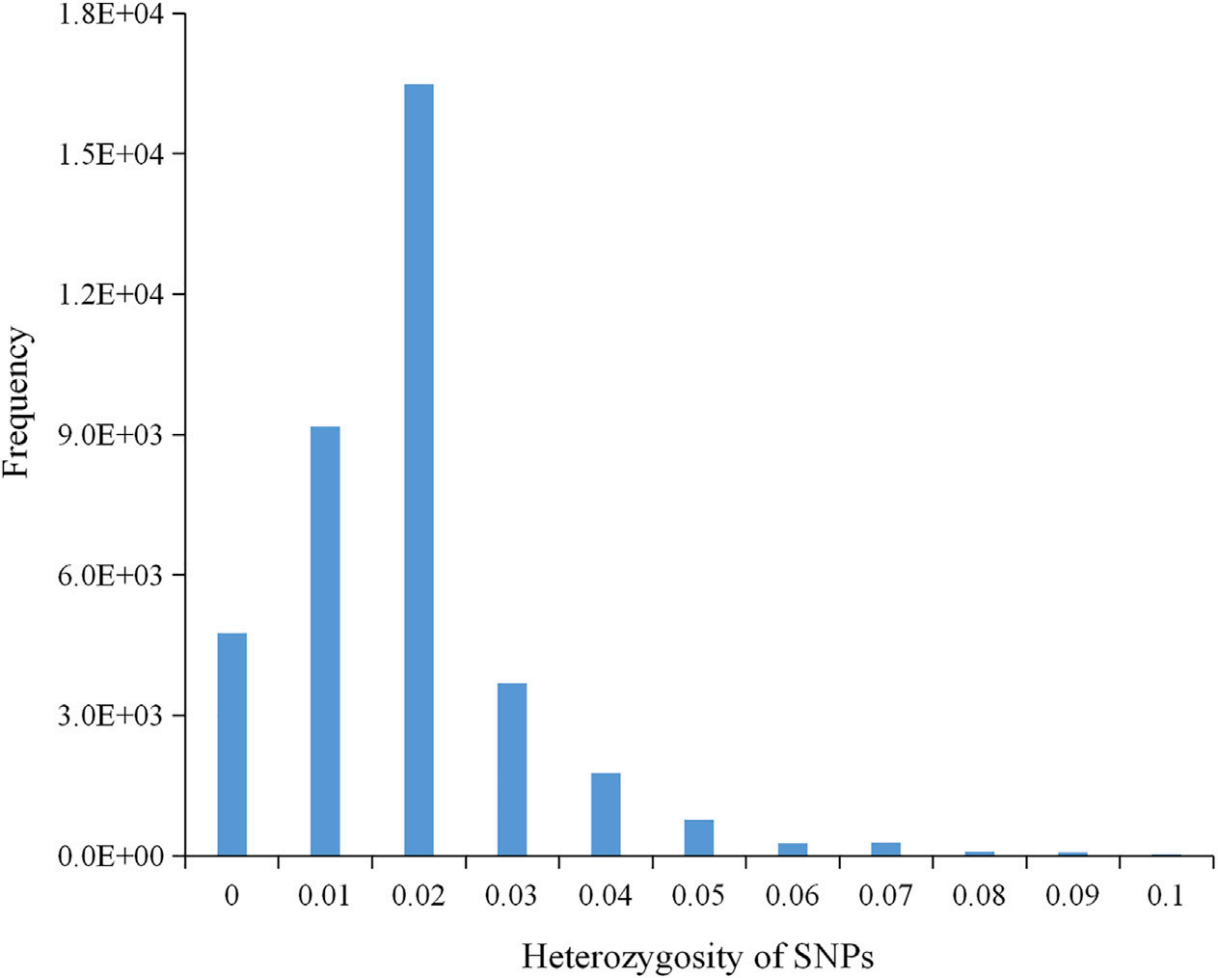
A histogram showing the normal distribution of heterozygosity of 37,929 SNPs.

**FIGURE 2:**
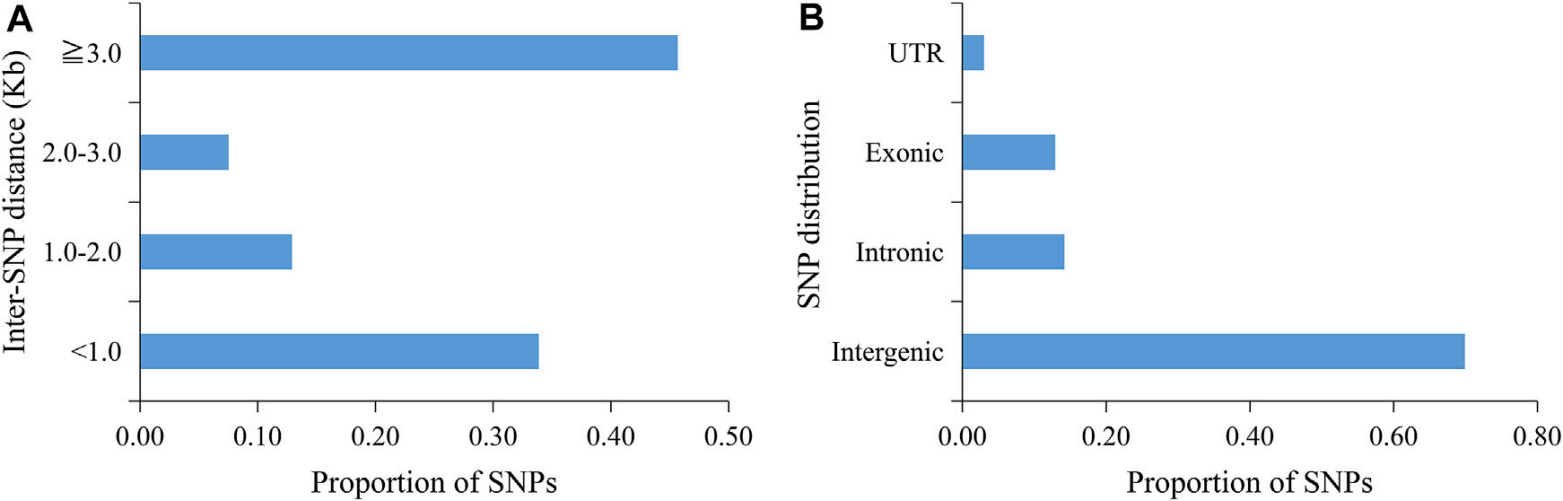
Genome-wide SNP density and distribution of 37,929 SNPs. **(A)** Interval statistics between SNPs. **(B)** Illustration of the ratio of SNPs in the intergenic region and different positions in the gene region.

A total of 699.14 Gb of raw data was generated by whole-genome sequencing 122 rice varieties, with an average of 5,730.7 Mb of data per sample. After filtration, 697.56 Gb of clean data was recovered, with an average of 5,717.7 Mb per sample; Q20 (the base call accuracy is 99%) was greater than 96%, and Q30 (the base call accuracy is 99.9%) was greater than 91% (Supplementary Table S3). On the basis of sequencing, a total of 37,929 SNPs were then obtained by alignment to the reference genome. Most of the SNP loci were low heterozygosity (Figure 1) and uniformly distributed in the genome with an average distribution density from 6.20 to 20.26 kb/SNP (Table 3; Supplementary Figure S1). For these SNPs, 33.87% of the inter-loci distances were in less than 1kb, and 45.68% were in more than 3 kb (Figure 2A). In addition, most of the SNP loci were located in intergenic regions, and the rest were in introns, coding regions, and UTR regions (Figure 2B).

**TABLE 3:** Chromosome distribution of SNP loci used for calculating the molecular distance.

Chromosome	Chr. length (Mb)	Number of SNP	Density (kb/SNP)
Chr.1	44.36	3822	11.61
Chr.2	37.76	3366	11.22
Chr.3	39.69	1959	20.26
Chr.4	35.85	4582	7.82
Chr.5	31.24	1770	17.65
Chr.6	32.47	2490	13.04
Chr.7	30.28	2976	10.17
Chr.8	29.95	3517	8.52
Chr.9	24.76	1946	12.72
Chr.10	25.58	3412	7.50
Chr.11	31.78	5126	6.20
Chr.12	26.60	2963	8.98

### Diversity Analysis Based on SNP Loci

**FIGURE 3:**
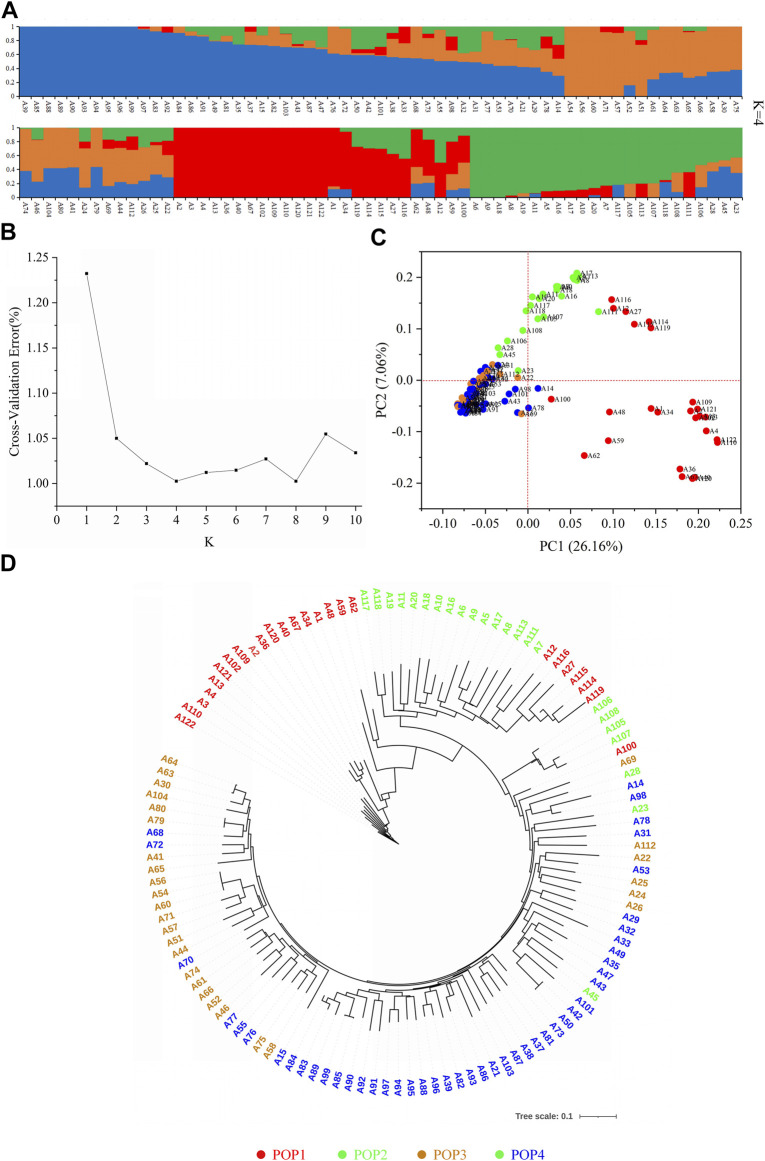
Details of population structure analysis by using 37,929 SNPs based on 122 rice genotypes. **(A)** Population structures. **(B)** Cross-validation error value of different subgroups. **(C)** Principal component analysis. **(D)** Evolutionary tree diagram. Red, green, orange, and blue represent POP1, POP2, POP3, and POP4, respectively.

Using Admixture software (Alexander et al., 2009), the genetic structure of 122 accessions was analyzed based on 37,929 SNPs. The results showed that the CVE showed a downward trend with an increase of the K value. When the K value was 4 and 8, the CVE reached the valley value (Figure 3B), and after further combination with PCA (Figure 3C), phylogenetic tree analysis (Figure 3D), and material source information, the tested materials were finally divided into four subgroups (Figure 3A). Among them, the composition of the POP1 subgroup was more complex, with 25 accessions from eight countries including China, Japan and the United States. The POP2 subgroup had 23 accessions, mostly from China. The POP3 subgroup had 28 accessions, mainly from Japan. The 46 accessions of the POP4 subgroup were mainly from Japanese breeding lines. According to the analysis results, varieties from the same country tended to be clustered together.

### Comparison of the Correlations Between Molecular and Phenotypic Distances of Rice Varieties by Different Similarity Algorithms

**FIGURE 4:**
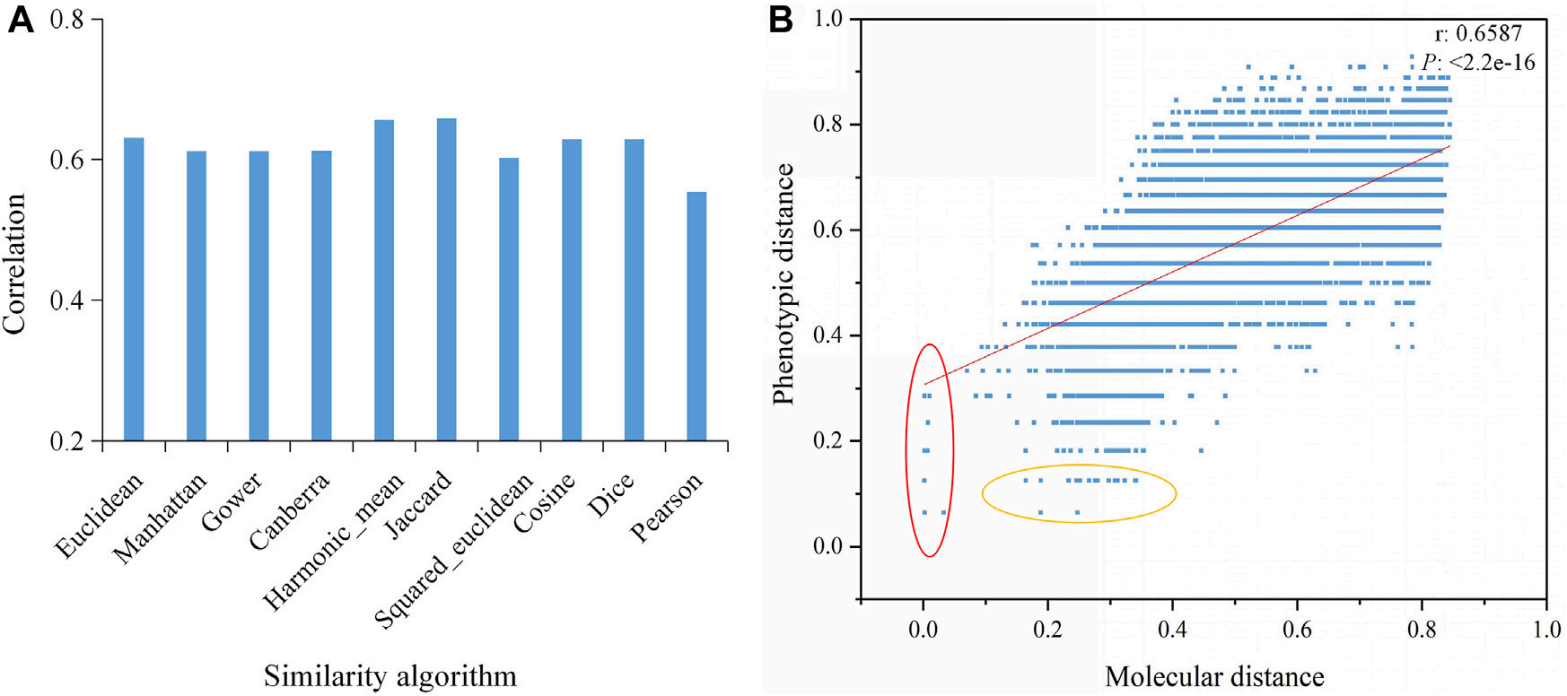
Correlations between the molecular and phenotypic distances based on different algorithms. **(A)** Correlations based on 10 similarity algorithms. **(B)** Correlation between the molecular and phenotypic distances of varieties based on the Jaccard algorithm. The red oval represents small molecular distances but large phenotypic distances, and the yellow oval represents small phenotypic distances but large molecular distances. *P* is the significance level, and r is the correlation coefficient.

We used 10 different similarity algorithms to analyze the correlation between the molecular and phenotypic distances of rice varieties and found that the algorithms had a significant impact on the correlations. Among them, the Jaccard algorithm had the highest correlation of 0.6587, whereas the correlation of the Pearson algorithm was only 0.5541 (Figure 4A). Furthermore, we found that some variety pairs showed small molecular distances but higher phenotypic distances, or small phenotypic distances but higher molecular distances (Figure 4B), suggesting that the phenotypic differences did not match the molecular differences. This might be an important reason for the low correlation.

### Effect of Numbers of SNP Loci and Phenotypic Traits on the Correlation Between Molecular and Phenotypic Distances

**FIGURE 5:**
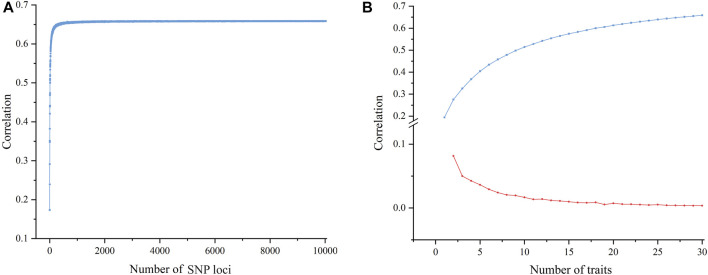
Scatter plot of the effect of different numbers of SNPs and traits on the correlation. **(A)** A scatter plot of the correlation between molecular and phenotypic distances shows that the correlation improves as the number of SNP loci increases until a ceiling is reached. **(B)** A scatter plot of correlation between molecular and phenotypic distances shows the correlation growth trend with an increasing number of traits; the blue line shows a gradual increase of correlation and the red line shows a reduced increase.

To investigate the factors that influence the correlation between molecular and phenotypic distances, we analyzed the effect of the numbers of SNP loci and phenotypic traits on the correlation by using the Jaccard algorithm. The results showed that as the number of SNP loci increased, the correlation increased rapidly at the beginning and became consistent at approximately 6.5; after that, the correlation did not change significantly even when the loci number continued to increase (Figure 5A). In terms of the number of phenotypic traits, there was also a plateau effect. The correlation initially increased with an increasing number of traits and then gradually leveled off (Figure 5B). The above results suggest that a certain number of SNP loci or phenotypic traits were enough to effectively improve the correlation between the molecular and phenotypic distances.

### Correlation Analysis of DUS Traits in Rice

**FIGURE 6:**
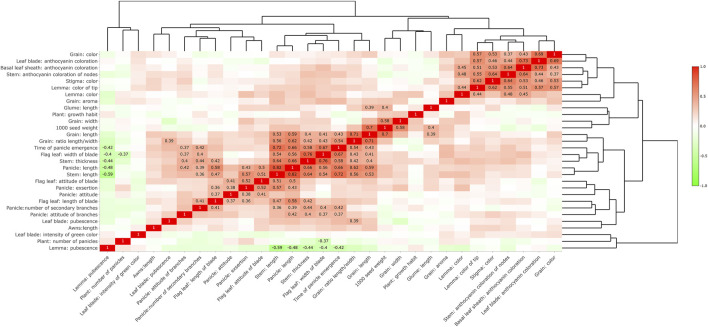
Correlation analysis of trait expression. Only values with a correlation greater than 0.35 or less than −0.35 are displayed.

Analysis results of the correlations of 30 DUS traits (Figure 6) showed that there was a positive correlation between the color of brown rice and the coloration of anthocyanins in the leaves, basal leaf sheaths, and stem nodes. Strong positive correlations were observed among grain length, grain aspect ratio, heading date, flag leaf width, stem length and thickness, and panicle length. The pubescence of the lemma was negatively correlated with the heading date, flag leaf width, stem length and thickness, and panicle length. The above results indicated that many phenotypic traits were closely related, and too strong a correlation might have a negative impact on the phenotypic clustering analysis of varieties.

### Genome-Wide Prediction Analysis of DUS Traits in Rice

To further analyze the effect of SNP loci on trait expression, we used the correlation coefficient between the predicted trait value and the actual phenotypic value as the standard of prediction accuracy. We used 37,929 SNP loci to predict 30 DUS traits (Table 4) with rrBLUP. The results showed that the prediction results of morphological traits were quite different, and the prediction accuracy ranged from 0.102 to 0.840, with an average of 0.479. Traits such as stem length and stem thickness showed an accuracy of over 0.8, and the accuracy of stem length was the highest at 0.840. Traits such as the basal sheath anthocyanin color, glume length, and intensity of green color of the leaf blade showed an accuracy of less than 0.2, and the accuracy of the intensity of green color of leaf blade was only 0.102.

**TABLE 4:** Correlation between predicted and true values of traits achieved by using rrBLUP.

No.	Trait	Correlation (predicted vs. measured traits)	Group	No.	Trait	Correlation (predicted vs. measured traits)	Group
1	Basal leaf sheath: anthocyanin coloration	0.129	A	16	Panicle: exsertion	0.608	B
2	Plant: growth habit	0.760	B	17	Glume: length	0.186	A
3	Leaf blade: intensity of green color	0.102	A	18	Lemma: color	0.540	B
4	Leaf blade: anthocyanin coloration	0.232	A	19	Grain: ratio length/width	0.565	B
5	Leaf blade: pubescence	0.447	B	20	Grain: color	0.360	A
6	Time of panicle emergence	0.765	B	21	Grain: aroma	0.287	A
7	Awn: length	0.307	A	22	Plant: number of panicles	0.315	A
8	Lemma: color of tip	0.384	A	23	Stem: thickness	0.817	B
9	Stigma: color	0.307	A	24	Stem: length	0.840	B
10	Stem: anthocyanin coloration of nodes	0.354	A	25	Flag leaf: length of blade	0.378	A
11	Lemma: pubescence	0.648	B	26	Flag leaf: width of blade	0.740	B
12	Flag leaf: attitude of blade	0.563	B	27	Panicle: length	0.760	B
13	Panicle: attitude	0.614	B	28	1000 seed weight	0.443	B
14	Panicle: number of secondary branches	0.441	A	29	Grain: length	0.605	B
15	Panicle: attitude of branches	0.439	A	30	Grain: width	0.433	A

**FIGURE 7:**
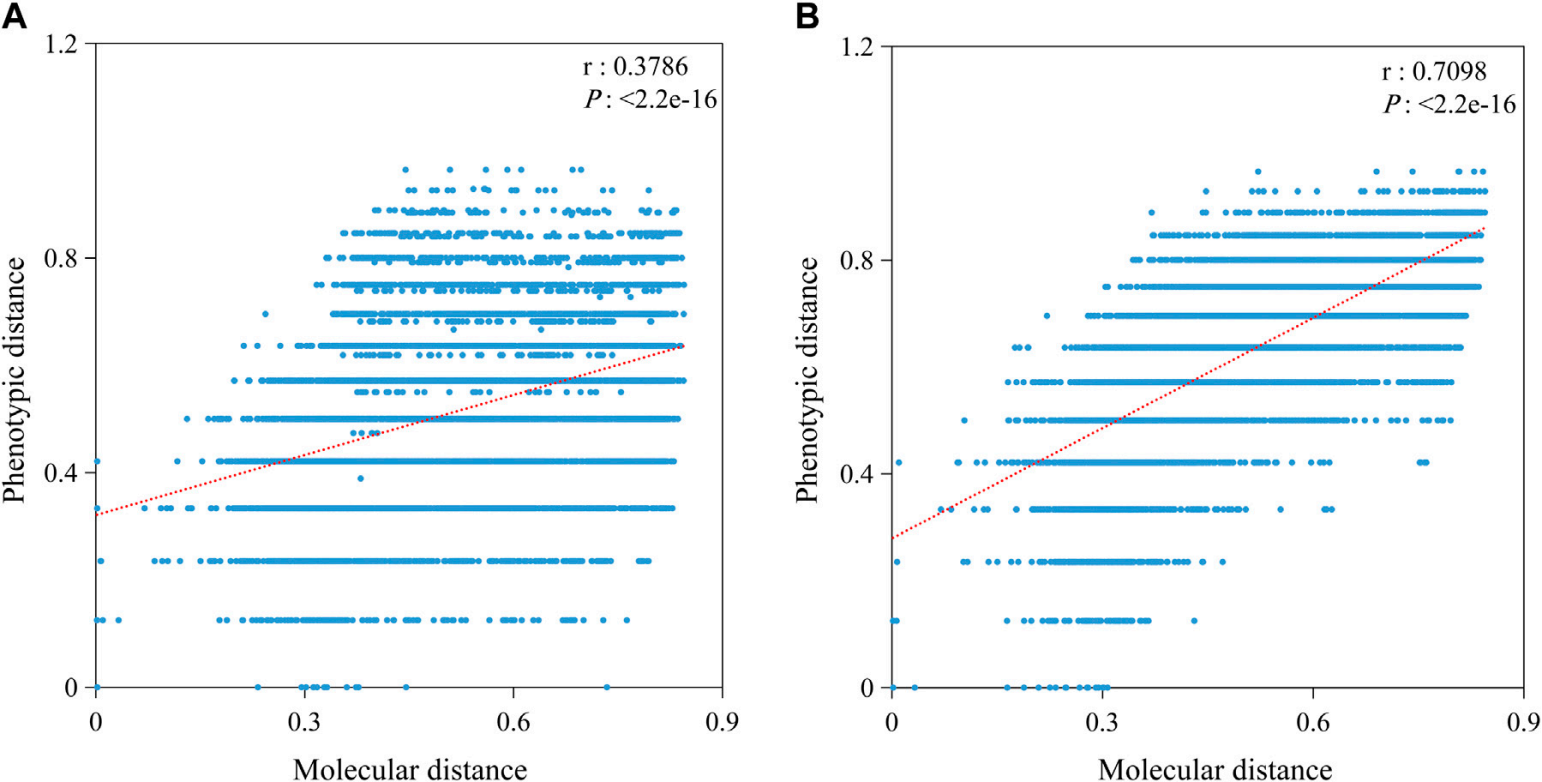
Correlation between molecular and phenotypic distances in different groups. **(A)** Correlation based on 15 traits with predicted values less than 0.443. **(B)** Correlation based on 15 traits with predicted values more than 0.443. *P* is the significance level, and r is the correlation coefficient.

To analyze the effect of different traits on the correlation, we divided the phenotypic traits into A and B groups with a prediction accuracy threshold of 0.443 (Table 4). The prediction accuracy of group A was less than 0.443, with an average of 0.310, and the prediction accuracy of group B was more than 0.443, with an average of 0.648. Furthermore, we performed correlation analysis between the molecular and phenotypic distances separately (Figure 7). The results showed that the correlation in group A (0.3786) was significantly less than that in group B (0.7098), suggesting that the key to improving the correlation between molecular and phenotypic distances of rice varieties was to improve the resolution of traits.

### Evaluation and Analysis of UPOV Option 2

**FIGURE 8:**
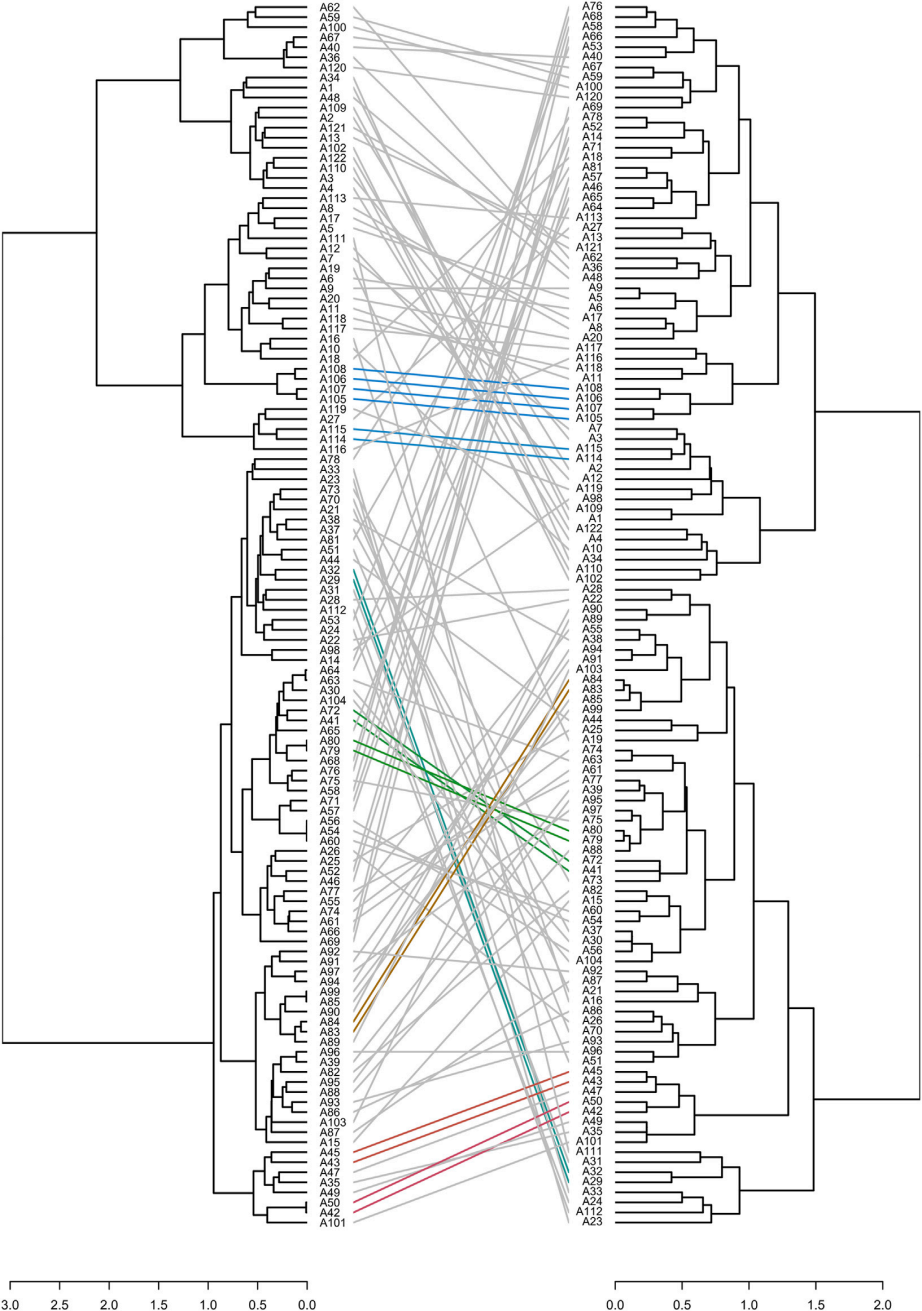
Correspondence between molecular (left of figure, calculated by using the Jaccard distance) and phenotypic (right of figure, calculated by using the Jaccard distance) cluster analysis. Identical colored lines indicate the same cluster results.

The key to UPOV option 2 is to reproduce the phenotype distinctness determination by setting molecular distance thresholds. As all varieties were phenotypically distinct from each other, we conducted distinctness determination analysis separately by setting different gradients of phenotypic distances and molecular distances, and we then counted the number of shared “D” varieties (phenotypically or molecularly distinct varieties according to artificially set distances). Finally, UPOV option 2 was evaluated based on the above method. The results (Table 5) showed that to identify 12 or 24 phenotypic “D” varieties, at least 72 molecular “D” varieties were needed, whereas to identify 48, 72, and 96 phenotypic “D” varieties, 122 molecular “D” varieties were needed. Furthermore, phenotypic and molecular clustering analyses were performed on the 122 varieties based on the Jaccard distance (Figure 8). The results showed that only nine pairs of cultivars (A29 and A32, A43 and A45, A42 and A50, A41 and A72, A79 and A80, A83 and A84, A105 and A107, A106 and A108, A114 and A115) had the same cluster analysis results. Among them, A79 and A80 were a pair of varieties from Japan, their molecular distances were very small, and the phenotypic differences were mainly reflected in the stem height and the attitude of the flag leaf blade. The phenotypic and molecular clustering results of A105, A106, A107, and A108 were the same; A107 and A108 were from the same breeding institutes. The phenotypic differences for the four varieties were mainly reflected in the heading date, stem length, and panicle length.

**TABLE 5:** Comparisons of distinctness decisions made by using either morphological or molecular distances.

Number of phenotypic “D” varieties	Number of molecular “D” varieties					
	12	24	48	72	96	122
12	4	7	11	12	12	12
24	5	13	23	24	24	24
48	12	23	40	45	47	48
72	12	24	47	60	69	72
96	12	24	48	66	83	96
122	12	24	48	72	96	122

“D” varieties are expressed as artificially set phenotypically or molecularly distinct varieties.

These results suggested that the determinations based on phenotypic distances and molecular distances were quite different, and phenotype distinctness testing could not be reproduced by setting molecular distance thresholds. Therefore, UPOV option 2 would not be sufficient for DUS testing in rice.

## Discussion

DUS testing is an important scientific basis for the authorization of new plant varieties. In order to improve the testing efficiency and quality, researchers have conducted in-depth studies on the correlation between molecular distances and phenotypic distances of varieties (Jones et al., 2013; Hong et al., 2021). Earlier reports showed that there was low correlation between phenotypic and molecular distances (Gupta et al., 2018; Guan et al., 2020), which might be related to the low number of molecular markers. With the development of sequencing technology and the reduction of sequencing costs, SNPs have become important molecular markers for diversity analysis. SNPs can be used to perform genome-wide association studies (Huang et al., 2012; Wang et al., 2020) and the rapid identification of high-throughput varieties (Yuan et al., 2022). In this study, based on the whole-genome resequencing of 122 rice germplasms, the screened 37,929 SNP loci were used to analyze the correlation between the molecular and phenotypic distances of rice varieties. The results showed that as the number of SNP loci increased, the correlation rapidly increased up to a level of approximately 6.5 and then entered a plateau phase. This finding indicated that although the number of SNP loci had an impact on the correlation, it could not be the most critical factor influencing the correlation. In addition, we also analyzed the effect of statistical algorithms on the correlation between the molecular and phenotypic distances. The results showed that relative to the other nine algorithms, the Jaccard similarity algorithm could achieve a higher correlation.

To decipher the ceiling effect of the correlation, we used the genome-wide prediction method to predict 30 phenotypic traits and found that the prediction accuracy of some traits, such as the basal sheath anthocyanin color, leaf blade anthocyanin color, stigma color, awn length, glume length, and intensity of green color of the leaf blade, was low. Furthermore, in combination with group comparison analysis, we found that the key to overcoming the correlation ceiling effect was to improve the resolution of low predictive value traits. In fact, we also screened SNPs near many known genes, such as Chr6_5311542 near the key anthocyanin regulator *OSC1* (Ithal and Reddy, 2004), Chr8_23986899 near the awn growth factor *GAD1* (Jin et al., 2016), and Chr5_16510158 near the chlorophyll synthase *YGL1* (Wu et al., 2007). However, the phenotype prediction effect of these SNPs in the above traits was not ideal. The reason for this problem was not only related to the low heritability of some traits (Jones and Mackay, 2015) but also to the expression state setting of some traits. For example, the setting of the expression state of the anthocyanin color in the basal leaf sheath was not linear, including both the degree of anthocyanin deposition and the presence or absence of purple lines. Therefore, it is necessary to further analyze this in future research.

The purpose of UPOV option 2 is to reproduce phenotypic distinctness determinations by calibrating molecular distances. Therefore, a high correlation between molecular and phenotypic distances is the key to implementing this option. Jones et al. (2013) found that when the correlation was lower than 0.6, the distinctness determination using the phenotypic distance differed by 80% compared to that using the molecular distance. Our study also found that even when the correlation reached 0.6587, there was still a large difference in the determination results. Therefore, at the current research level, the phenotypic and molecular distances cannot match perfectly, and UPOV option 2 is not able to replace the traditional phenotypic DUS testing for the time being (Guan et al., 2020). However, we also found that the genome-wide prediction method could be used to predict some traits more accurately. Therefore, in order to improve the application level of UPOV option 2, the whole-genome prediction method should be combined into the option. On the other hand, with the rapid reduction of sequencing costs, large numbers of SNP loci are being continually developed, and UPOV options 1 and 3 have also attracted much attention. For UPOV option 1, the functional marker *Pi54* *MAS* was used to improve the rice blast-resistant restorer line (Ramalingam et al., 2020). Selection analysis was conducted for rice grain size based on the novel functional markers of 14 genes (Zhang et al., 2020). A new mutation site was identified through sequence analysis of the rice *SD1* gene. On this basis, a new functional molecular marker for marker-assisted selection was developed by Bhuvaneswari et al. (2020). Since the current development of functional molecular markers in rice mainly focuses on important agronomic traits such as yield, quality, and resistance, and there are few studies on other non-major agronomic traits, the application of UPOV option 1 in rice variety distinctness testing has not yet been reported. In addition, there may also be a certain relationship between the effect of functional molecular markers and the genetic background of the material. Studies have shown that there is a close linkage between the color of the apiculus and stigma in rice (Zhao et al., 2016; Tong et al., 2021). However, Zhao et al. (2016) transferred the chromogen for anthocyanin *OSC1* to the *japonica* variety Kitaake (white apiculus and stigma) and found that the apiculus of the transgenic plant exhibited red coloration but the stigma was achromatic. Therefore, the combination of UPOV options 1 and 2 for DUS testing is of great significance for the development of molecular identification technology.

For option 3, although variety authorization can be completed within a few weeks by using this option, the distinctness of a variety defined by molecular markers is meaningless if the variety is not phenotypically unique. In addition, for rice varieties, it is normal and acceptable to have a certain number of off-type plants. If molecular markers are used for uniformity testing, it will be hard to evaluate the heterogeneity (Xu, 2014). Therefore, to establish a test system based entirely on molecular markers, it is necessary to fully consider the influences of various factors such as traits, distinctness thresholds, variety protection purposes, and sampling methods. This is why there is much controversy (UPOV INF/18/1, 2011) about UPOV option 3.

## Conclusion

In this study, based on the whole-genome resequencing of 122 rice accessions, the 37,929 SNP loci screened were used to analyze the correlation between the molecular and phenotypic distances of rice varieties, and UPOV option 2 was also evaluated. The results showed that statistical algorithms, the number of phenotypic traits, and the number of SNP loci all affected the correlation between the molecular and phenotypic distances of the rice varieties. Among the statistical algorithms, the Jaccard similarity algorithm had the highest correlation of 0.6587. In terms of the number of SNP loci and phenotypic traits, we found that the correlation between the molecular and phenotypic distances had a ceiling effect, and the ceiling effect for the number of SNPs was more obvious. Furthermore, to overcome the ceiling effect of correlation, we predicted 30 DUS traits by using genome-wide prediction and performed a comparative analysis based on prediction accuracy. The results suggested that improving the resolution of traits with low predictive value might be the key to overcoming the ceiling effect of correlation. In addition, we also used molecular distances and phenotypic distances to analyze the distinctness of rice varieties, and we found that the results of the two methods were quite different, indicating that UPOV option 2 could not be used alone for DUS testing, whereas genotype and phenotype analysis together could improve the efficiency of DUS testing.

## Data Availability

The original contributions presented in this study are included in the article/Supplementary Material; further inquiries can be directed to the corresponding authors.

## Supplementary Material

Supplementary Figure S1 Genome-wide SNP density plot.

## References

[B1] Alexander D. H. Novembre J. Lange K. (2009). Fast model-based estimation of ancestry in unrelated individuals. Genome Res. 19 (9), 1655–1664. 10.1101/gr.094052.109 19648217 PMC2752134

[B2] Bhuvaneswari S. Krishnan S. G. Ellur R. K. Vinod K. K. Bollinedi H. Bhowmick P. K. (2020). Discovery of a novel induced polymorphism in *SD1* gene governing semi-dwarfism in rice and development of a functional marker for marker-assisted selection. Plants 9 (9), 1198. 10.3390/plants9091198 PMC7570060 32937792

[B3] Cockram J. Jones H. Norris C. O'Sullivan D. M. (2012). Evaluation of diagnostic molecular markers for DUS phenotypic assessment in the cereal crop, barley (*Hordeum vulgare* ssp. *vulgare* L.). Theor. Appl. Genet. 125 (8), 1735–1749. 10.1007/s00122-012-1950-3 22898724

[B4] Cui Y. Li R. Li G. Zhang F. Zhu T. Zhang Q. (2020). Hybrid breeding of rice via genomic selection. Plant Biotechnol. J. 18 (1), 57–67. 10.1111/pbi.13170 31124256 PMC6920338

[B5] Danecek P. Auton A. Abecasis G. Albers C. A. Banks E. DePristo M. A. (2011). The variant call format and VCF tools. Bioinformatics 27 (15), 2156–2158. 10.1093/bioinformatics/btr330 21653522 PMC3137218

[B6] Dodia S. M. Joshi B. Gangurde S. S. Thirumalaisamy P. P. Mishra G. P. Narandrakumar D. (2019). Genotyping-by-sequencing based genetic mapping reveals large number of epistatic interactions for stem rot resistance in groundnut. Theor. Appl. Genet. 132 (4), 1001–1016. 10.1007/s00122-018-3255-7 30539317

[B7] Drost H. G. (2018). Philentropy: Information theory and distance quantification with R. J. Open Source Softw. 3 (26), 765. 10.21105/joss.00765

[B8] Ebana K. Yonemaru J. Fukuoka S. Iwata H. Kanamori H. Namiki N. (2010). Genetic structure revealed by a whole-genome single-nucleotide polymorphism survey of diverse accessions of cultivated Asian rice (*Oryza sativa* L.). Breed. Sci. 60, 390–397. 10.1270/jsbbs.60.390

[B9] Endelman J. B. (2011). Ridge regression and other kernels for genomic selection with R package rrBLUP. Plant Genome 4, 250–255. 10.3835/plantgenome2011.08.0024

[B10] Food and Agriculture Organization (2020). Data from: Crops and livestock products. Rome: FAOSTAT. https://www.fao.org/faostat/en/#data/QCL.

[B11] Galili T. (2015). Dendextend: an R package for visualizing, adjusting and comparing trees of hierarchical clustering. Bioinformatics 31 (22), 3718–3720. 10.1093/bioinformatics/btv428 26209431 PMC4817050

[B12] Gangurde S. S. Ghorade R. B. Moharil M. P. Ingle K. P. Wagh A. (2017). Microsatellite based DNA fingerprinting of sorghum [*Sorghum bicolor* (L.) Moench] hybrid CSH-35 with its parents. bioscan 12 (1), 215–219.

[B13] Gangurde S. S. Wang H. Yaduru S. Pandey M. K. Fountain J. C. Chu Y. (2020). Nested-association mapping (NAM) ‐based genetic dissection uncovers candidate genes for seed and pod weights in peanut (*Arachis hypogaea*). Plant Biotechnol. J. 18 (6), 1457–1471. 10.1111/pbi.13311 31808273 PMC7206994

[B14] Guan J. J. Zhang P. Huang Q. M. Wang J. M. Yang X. H. Chen Q. B. (2020). SNP markers potential applied in DUS testing of maize. Int. J. Agric. Biol. 23, 417–422. 10.17957/IJAB/15.1304

[B15] Gupta S. K. Nepolean T. Shaikh C. G. Rai K. Hash C. T. Das R. R. (2018). Phenotypic and molecular diversity-based prediction of heterosis in pearl millet (*Pennisetum glaucum* L. (R.) Br.). Crop J. 6 (3), 271–281. 10.1016/j.cj.2017.09.008

[B16] Hayward A. C. Tollenaere R. Dalton Morgan J. Batley J. (2015). Molecular marker applications in plants. Methods Mol. Biol. 1245, 13–27. 10.1007/978-1-4939-1966-6_2 25373746

[B17] Hong Y. Pandey M. K. Lu Q. Liu H. Gangurde S. S. Li S. (2021). Genetic diversity and distinctness based on morphological and SSR markers in peanut. Agron. J. 113 (6), 4648–4660. 10.1002/agj2.20671

[B18] Hu P. Zhai H. Wan J. (2002). New characteristics of rice production and quality improvement in China. Rev. China Agric. Sci. Technol. 4 (4), 33–39. in Chinese with English abstract. 10.3969/j.issn.1008-0864.2002.04.006

[B19] Huang X. Zhao Y. Wei X. Li C. Wang A. Zhao Q. (2012). Genome-wide association study of flowering time and grain yield traits in a worldwide collection of rice germplasm. Nat. Genet. 44, 32–39. 10.1038/ng.1018 22138690

[B20] Ithal N. Reddy A. R. (2004). Rice flavonoid pathway genes, *OsDfr* and *OsAns*, are induced by dehydration, high salt and ABA, and contain stress responsive promoter elements that interact with the transcription activator, OsC1-MYB. Plant Sci. 166 (6), 1505–1513. 10.1016/j.plantsci.2004.02.002

[B21] Jadhav M. P. Gangurde S. S. Hake A. A. Yadawad A. Mahadevaiah S. S. Pattanashetti S. K. (2021). Genotyping-by-sequencing based genetic mapping identified major and consistent genomic regions for productivity and quality traits in peanut. Front. Plant Sci. 12, 668020. 10.3389/fpls.2021.668020 34630444 PMC8495222

[B22] Jin J. Hua L. Zhu Z. Tan L. Zhao X. Zhang W. (2016). *GAD1* encodes a secreted peptide that regulates grain number, grain length, and awn development in rice domestication. Plant Cell 28 (10), 2453–2463. 10.1105/tpc.16.00379 27634315 PMC5134979

[B48] Jones H. Mackay I. (2015). Implications of using genomic prediction within a high-density SNP dataset to predict DUS traits in barley. Theor. Appl. Genet. 128 (12), 2461–2470. 10.1007/s00122-015-2601-2 26350495

[B23] Jones H. Norris C. Smith D. Cockram J. Lee D. O’Sullivan D. M. (2013). Evaluation of the use of high-density SNP genotyping to implement UPOV model 2 for DUS testing in barley. Theor. Appl. Genet. 126 (4), 901–911. 10.1007/s00122-012-2024-2 23232576

[B24] Liu C. Zhang G. (2010). SSR analysis of genetic diversity and the temporal trends of major commercial inbred *indica* rice cultivars in South China in 1949-2005. Acta Agron. Sin. 36 (11), 1843–1852. in Chinese with English abstract. 10.1016/s1875-2780(09)60082-1

[B25] Liu H. Xu Z. Rao D. Lu Q. Li S. Liu H. (2019). Genetic diversity analysis and distinctness identification of peanut cultivars based on morphological traits and SSR markers. Acta Agron. Sin. 45 (1), 26–36. in Chinese with English abstract. 10.3724/SP.J.1006.2019.84060

[B26] McKenna A. Hanna M. Banks E. Sivachenko A. Cibulskis K. Kernytsky A. (2010). The genome analysis toolkit: A mapreduce framework for analyzing next-generation DNA sequencing data. Genome Res. 20 (9), 1297–1303. 10.1101/gr.107524.110 20644199 PMC2928508

[B27] Ministry of Agriculture and Rural Affairs of People’s Republic of China (2021). Data from: China seed industry big data platform. Beijing: CSIBDP. http://202.127.42.145/bigdataNew/home/service.

[B28] Pourabed E. Noushabadi M. R. J. Jamali S. H. Alipour N. M. Zareyan A. Sadeghi L. (2015). Identification and DUS testing of rice varieties through microsatellite markers. Int. J. Plant Genomics 2015, 965073. 10.1155/2015/965073 25755666 PMC4337753

[B29] Pujar M. Gangaprasad S. Govindaraj M. Gangurde S. S. Kanatti A. Kudapa H. (2020). Genome-wide association study uncovers genomic regions associated with grain iron, zinc and protein content in pearl millet. Sci. Rep. 10, 19473. 10.1038/s41598-020-76230-y 33173120 PMC7655845

[B30] R Core Team (2012). Team RDC.R: A language and environment for statistical computing. Vienna, Austria: R foundation for statistical computing.

[B31] Ramalingam J. Palanisamy S. Alagarasan G. Renganathan V. G. Ramanathan A. Saraswathi R. (2020). Improvement of stable restorer lines for blast resistance through functional marker in rice (*Oryza sativa* L.). Genes 11, 1266. 10.3390/genes11111266 PMC7692511 33121205

[B32] Shasidhar Y. Variath M. T. Vishwakarma M. K. Manohar S. S. Gangurde S. S. Sriswathi M. (2020). Improvement of three popular Indian groundnut varieties for foliar disease resistance and high oleic acid using SSR markers and SNP array in marker-assisted backcrossing. Crop J. 8 (1), 1–15. 10.1016/j.cj.2019.07.001

[B33] Steele K. Tulloch M. Q. Burns M. Nader W. (2021). Developing KASP markers for identification of basmati rice varieties. Food Anal. Methods 14, 663–673. 10.1007/s12161-020-01892-3

[B34] The 3K RGP (2014). The 3000 rice genomes project. GigaScience 3, 7. 10.1186/2047-217X-3-7 24872877 PMC4035669

[B35] Tong J. Han Z. Han A. (2021). Mapping of quantitative trait loci for purple stigma and purple apiculus in rice by using a Zhenshan 97B/Minghui 63 RIL population. Czech J. Genet. Plant Breed. 57, 113–118. 10.17221/20/2021-CJGPB

[B36] UPOV INF/18/1 (2011). Guidance on the use of biochemical and molecular makers in the examination of distinctness, uniformity and stability (DUS). Geneva: International Union for the Protection of New Varieties of Plants. https://www.upov.int/edocs/infdocs/en/upov_inf_18.pdf .

[B37] UPOV TG/16/8 (2004). Guidelines for the conduct of tests for distinctness, uniformity and stability of rice. Geneva: International Union for the Protection of New Varieties of Plants. http://www.upov.org/en/publications/tg-rom/tg016/tg_16_8.pdf .

[B38] UPOV TG/16/9 (2020). Guidelines for the conduct of tests for distinctness, uniformity and stability of rice. Geneva: International Union for the Protection of New Varieties of Plants. https://www.upov.int/edocs/tgdocs/en/tg016.pdf .

[B39] Wagh S. G. Pohare M. B. Kale R. R. (2021). Chapter 9-CRISPR/Cas in food security and plant disease management. Food Secur. Plant Dis. Manag., 171–191. 10.1016/B978-0-12-821843-3.00020-9

[B47] Wang Q. Tang J. Han B. Huang X. (2020). Advances in genome-wide association studies of complex traits in rice. Theor. Appl. Genet. 133 (5), 1415–1425. 10.1007/s00122-019-03473-3 31720701

[B40] Wu Z. Zhang X. He B. Diao L. Sheng S. Wang J. (2007). A chlorophyll-deficient rice mutant with impaired chlorophyllide esterification in chlorophyll biosynthesis. Plant Physiol. 145 (1), 29–40. 10.1104/pp.107.100321 17535821 PMC1976586

[B41] Xu Y. (2014). Molecular Plant Breeding (in Chinese). Beijing: Science Press.

[B42] Yang J. Lee S. H. Goddard M. E. Visscher P. M. (2011). Gcta: A tool for genome-wide complex trait analysis. Am. J. Hum. Genet. 88, 76–82. 10.1016/j.ajhg.2010.11.011 21167468 PMC3014363

[B43] Yuan X. Li Z. Xiong L. Song S. Zheng X. Tang Z. (2022). Effective identification of varieties by nucleotide polymorphisms and its application for essentially derived variety identification in rice. BMC Bioinforma. 23, 30. 10.1186/s12859-022-04562-9 PMC8751067 35012448

[B44] Zhang L. Ma B. Bian Z. Li X. Zhang C. Liu J. (2020). Grain size selection using novel functional markers targeting 14 genes in rice. Rice 13, 63. 10.1186/s12284-020-00427-y 32902771 PMC7481322

[B45] Zhao S. Wang C. Ma J. Wang S. Tian P. Wang J. (2016). Map-based cloning and functional analysis of the chromogen gene *C* in rice (*Oryza sativa* L.). J. Plant Biol. 59, 496–505. 10.1007/s12374-016-0227-9

[B46] Zheng X. H. Ye J. H. Cheng C. P. Wei X. H. Ye X. F. Yang Y. L. (2022). *Xian-geng* identification by SNP markers in *Oryza sativa* L. Acta Agron. Sin. 48 (2), 342–352. in Chinese with English abstract. 10.3724/SP.J.1006.2022.02085

