# Supplementary figures and images for: Whole Genome Sequencing and Morphological Trait-Based Evaluation of UPOV Option 2 for DUS Testing in Rice
Source: Front Genet. 2022 Aug 26;13:945015. doi: 10.3389/fgene.2022.945015 (PMC9458885; doi:10.3389/fgene.2022.945015)

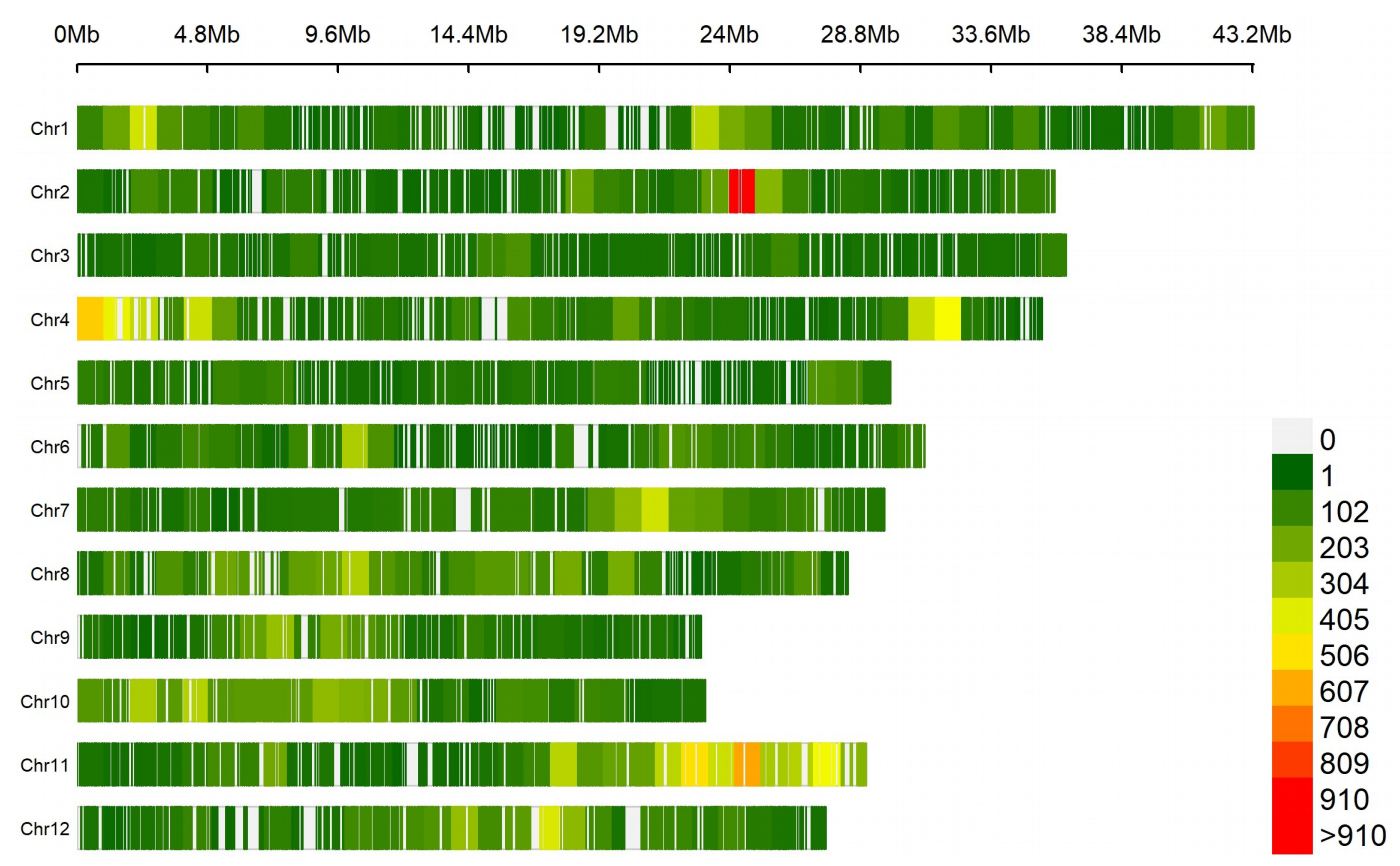

Supplement: Supplementary file 3 [file Image1.TIF]
